# Baicalein attenuates bleomycin-induced lung fibroblast senescence and lung fibrosis through restoration of Sirt3 expression

**DOI:** 10.1080/13880209.2022.2160767

**Published:** 2023-02-23

**Authors:** Yuan Ji-hong, Ma Yu, Yuan Ling-hong, Gong Jing-jing, Xu Ling-li, Wang Lv, Jin Yong-mei

**Affiliations:** a Department of Acute and Critical Care, Shanghai Seventh People’s Hospital of Shanghai University of Traditional Chinese Medicine, Shanghai, China; b Department of Anesthesiology, Shanghai Baoshan Traditional Chinese Medicine-integrated Hospital, Shanghai, China; c Department of Acute and Critical Care, Changxing Branch of Xinhua Hospital Affiliated to School of Medicine, Shanghai Jiaotong University, Shanghai, China; d Department of Nephrology, Shanghai Seventh People’s Hospital of Shanghai University of Traditional Chinese Medicine, Shanghai, China; e Department of Emergency and Critical Care Medicine, Shanghai Changzheng Hospital, Naval Medical University, Shanghai, China; f Department of Nursing, Shanghai Seventh People’s Hospital of Shanghai University of Traditional Chinese Medicine, Shanghai, China

**Keywords:** TGF-β1, idiopathic pulmonary fibrosis, Smad

## Abstract

**Context:**

Fibroblast senescence was reported to contribute to the pathological development of idiopathic pulmonary fibrosis (IPF), and baicalein is reported to attenuate IPF.

**Objective:**

This study explores whether baicalein attenuates lung fibrosis by regulating lung fibroblast senescence.

**Materials and methods:**

Institute of Cancer Research (ICR) mice were randomly assigned to control, bleomycin (BLM), baicalein and BLM + baicalein groups. Lung fibrosis was established by a single intratracheal dose of BLM (3 mg/kg). The baicalein group received baicalein orally (100 mg/kg/day). Sirtuin 3 (Sirt3) siRNA (50 μg) was injected through the tail vein once a week for 2 weeks to explore its effect on the anti-pulmonary fibrosis of baicalein.

**Results:**

BLM-treated mice exhibited obvious lung fibrosis and fibroblast senescence by showing increased levels of collagen deposition (27.29% vs. 4.14%), hydroxyproline (208.05 vs. 40.16 ng/mg), collagen I (25.18 vs. 9.15 μg/mg), p53, p21, p16, MCP-1, PAI-1, TNF-α, MMP-10 and MMP-12 in lung tissues, which were attenuated by baicalein. Baicalein also mitigated BLM-mediated activation of TGF-β1/Smad signalling pathway. Baicalein restored the BLM-induced downregulation of Sirt3 expression in lung tissues and silencing of Sirt3 abolished the inhibitory role of baicalein against BLM-induced lung fibrosis, fibroblast senescence and activation of TGF-β1/Smad signalling pathway.

**Conclusions:**

Baicalein preserved the BLM-induced downregulation of lung Sirt3 expression, and thus the suppression of TGF-β1/Smad signalling pathway and lung fibrosis, which might provide an experimental basis for treatment of IPF.

## Introduction

Idiopathic pulmonary fibrosis (IPF), the most common form of idiopathic interstitial pneumonia, causes progressive pulmonary fibrosis, which is characterized by repeated epithelial cell damage, destruction of alveolar structure and pro-fibrotic mediator-induced extracellular matrix (ECM) deposition by myofibroblasts. The occurrence and factors associated with IPF include genetic susceptibility and other risk factors, such as bacterial or viral infection, smoking and environmental pollution (Cao et al. 2021). IPF has a high mortality rate, with the average survival time being 2–4 years from diagnosis (Wolters et al. 2014). Currently, only pirfenidone and nintedanib are approved by the Food and Drug Administration (FDA) (Ma et al. 2022) and recommended in the recent ATS/ERS/JRS/ALAT clinical practice guideline for the treatment of IPF (Raghu et al. 2022). Although the clinical trials with pirfenidone and nintedanib in IPF were reported to reduce the decline in FVC compared to placebo and the antifibrotic therapies are associated with improved survival in patients with IPF, this therapy could not completely stop the deterioration of lung function over time (Ma et al. 2022; Takehara et al. 2022). Therefore, identifying effective therapeutic methods for IPF is urgent.

The formation of fibrotic foci in IPF is mainly due to excessive ECM protein deposition in the alveolar space, with activated myofibroblasts being the main producers of pulmonary ECM (Du S-F et al. 2019; Blokland et al. 2021; Chen T et al. 2021; Wang Q et al. 2021; Zhang et al. 2021; Röhrich et al. 2022; Wu et al. 2022). Wu et al. (2022) demonstrated that the expression of checkpoint kinases 1 and 2 (CHK1/2) was markedly increased in the lungs, remodelled pulmonary arteries and isolated fibroblasts from IPF patients and animal models. CHK1/2 inhibition could interfere with TGF-β1-mediated fibroblast activation, attenuating fibrosis and pulmonary vascular remodelling. Zhang et al. (2021) indicated that N-methyladenosine modification expression was upregulated in a bleomycin (BLM)-induced pulmonary fibrosis mouse model, fibroblast-to-myofibroblast (FMT)-derived myofibroblasts and IPF patient lung samples. N^6^-methyladenosine (m^6^A) modification contributes to IPF-induced pulmonary fibrosis by regulating FMT. Of note, an increasing number of studies highlight the critical role of fibroblast senescence in the pathological process of IPF, as well as the existence of an increased and persistent number of senescent fibroblasts in lung tissues with IPF (Schafer et al. 2017). Senolytic drugs, including quercetin and dasatinib plus quercetin have been reported to attenuate IPF-induced pulmonary fibrosis and dysfunction in mice by selectively eliminating senescent fibroblasts and/or myofibroblasts (Hecker et al. 2014; Alvarez et al. 2017; Schafer et al. 2017; Hohmann et al. 2019), providing a potential therapeutic method for IPF.

Baicalein (5,6,7-trihydroxyflavone) is a major phenolic flavonoid extracted from the root of *Scutellaria baicalensis* Georgi (Lamiaceae) and widely used to treat multiple diseases, such as Alzheimer’s disease, Parkinson’s disease (Li Y et al. 2017), neuroinflammation (Rui et al. 2020), ischemia–reperfusion-induced brain injury (Yang et al. 2019), hyperuricaemia (Chen Y et al. 2021), sepsis-induced liver injury (Liu A et al. 2015), avian pathogenic *Escherichia coli*-induced acute lung injury, pulmonary arterial hypertension (Shi et al. 2018) and pulmonary fibrosis (Gao et al. 2013; Sun X et al. 2020), due to its anti-inflammatory, antioxidant and anti-apoptosis effects. Gao et al. (2013) reported that baicalein mitigated lung fibrosis in a rat model of IPF by suppressing miRNA (miR)-21 and TGF-β/Smad signalling. Sun et al. (2020) showed that baicalein attenuated TGF-β1-mediated collagen production in lung fibroblasts by inhibiting the expression of connective tissue growth factor.

In the present study, a mouse model of BLM-induced IPF was used to explore the protective effects of baicalein. Baicalein could inhibit BLM-induced lung fibroblast senescence and pulmonary fibrosis by suppressing the TGF-β1/Smad signalling pathway. It was also found that baicalein markedly increased sirtuin 3 (Sirt3) expression in the lung tissue of BLM-treated mice, which may contribute to the inhibitory effects of baicalein against BLM-induced lung fibroblast senescence and pulmonary fibrosis.

## Materials and methods

### Animals and drug administration

Institute of Cancer Research (ICR) mice (male; 8 weeks old) were provided by Shanghai SLAC Laboratory Animal Co. (Shanghai, China) and provided with free access to food and water in a controlled temperature of 23–25 °C. All animal protocols were approved by the Ethics Committee of the Experimental Animals of Shanghai Seventh People’s Hospital (approval no. 2020-AR-053). Mice were anesthetized using sodium pentobarbital (i.p., 30 mg/kg; MilliporeSigma, Burlington, MA) and then subjected to a single intratracheal dose of BLM (3 mg/kg; Selleck Chemicals, Houston, TX) diluted in 50 µL sterile saline. Mice that had been intratracheally administrated 50 µL sterile saline served as the control. In the baicalein group, mice received baicalein orally (100 mg/kg/day; MilliporeSigma, Burlington, MA); the dose was based on a previous study, which showed that baicalein (p.o. 100 mg/kg/day) markedly mitigated BLM-induced lung fibrosis (Gao et al. 2013).

### Experimental groups and drug treatment

The first experiment was performed to explore the effect of baicalein on BLM-mediated pulmonary fibrosis. Mice were randomly divided into four groups: (i) control group (*n* = 7), mice were intratracheally administrated sterile saline; (ii) BLM group (*n* = 7), mice were intratracheally administrated BLM; (iii) baicalein group (*n* = 7), mice were administrated saline intratracheally and baicalein orally; and (iv) BLM + baicalein group (*n* = 7), mice were administrated BLM intratracheally and baicalein orally. Mice were sacrificed after 2 weeks of BLM and baicalein administration.

The second experiment aimed to examine whether Sirt3 siRNA could abolish the protective role of baicalein against BLM-induced pulmonary fibrosis. Mice were randomly divided into six groups: (i) control group (*n* = 7); (ii) BLM group (*n* = 7); (iii) BLM + baicalein group (*n* = 7); (iv) Sirt3 siRNA group (*n* = 7); (v) BLM + Sirt3 siRNA group (*n* = 7); and (vi) BLM + baicalein + Sirt3 siRNA group (*n* = 7). Following drug administration, mice in the control siRNA group were injected with 50 μg control siRNA and those in the Sirt3 siRNA group were injected 50 μg Sirt3 siRNA through the tail vein once a week for 2 weeks.

### Sirt3 siRNA administration

In vivo-jetPEI™ was used for control and Sirt3 siRNA administration, according to the manufacturer’s instructions. Briefly, control or Sirt3 siRNA (50 μg) was dissolved in a 100-µL mixture of equal volumes of in vivo-jetPEI™ and 10% glucose, and then injected into the tail vein once a week for 2 weeks. The Sirt3 siRNAs were designed and synthesized by Shanghai GenePharma Co., Ltd. (Shanghai, China) and the sequences were as follows: Sirt3 siRNA sense, 5′-GUCUGAAGCAGUACAGAAAtt-3′ and antisense, 5′-UUUCUGUACUGCUUCAGACaa-3′ (Srivastava et al. 2018); control siRNA sense, 5′-UUCUCCGAACGUGUCACGUTT-3′ and antisense, 5′-ACGUGACACGUUCGGAGAATT-3′ antisense (Tang et al. 2018).

### Masson’s trichrome staining

The left lower pulmonary tissues were fixed in paraformaldehyde and then dehydrated in graded alcohol and embedded in paraffin. Paraffin-embedded sections (5 µm) were subjected to Masson’s trichrome staining (Wuhan Servicebio Technology Co., Ltd., Wuhan, China) to measure the fibrotic areas, according to the manufacturer’s instructions (Du S-F et al. 2019). Image-Pro Plus software version 6.0 (Media Cybernetics, Inc., Rockville, MD) was used to quantify the fibrotic area by manually examining the blue area (Du S-F et al. 2019). The researcher examining the fibrotic area was blinded to group allocation.

### Measurement of hydroxyproline and collagen I content

Cold PBS containing proteinase inhibitor cocktail (Sigma-Aldrich; Merck KgaA, Darmstadt, Germany) was used to homogenize pulmonary tissues, and hydroxyproline content (Winching, Nanjing, China) and collagen I (R&D Systems, Inc., Minneapolis, MN) were examined according to the manufacturer’s instructions.

### Isolation of mouse lung fibroblasts

Mouse pulmonary tissues were cut into pieces and then digested for 90 min as 37 °C with gentle shaking. Dulbecco’s modified Eagle’s medium-prepared digestion solution contained collagenase type III (0.1 U/mL, Worthington, Lakewood, NJ), trypsin (0.125%, Gibco; Thermo Fisher Scientific, Inc., Waltham, MA) and DNase I (0.1 U/mL, Thermo Fisher Scientific, Inc., Waltham, MA). Following filtration, cells were collected for subsequent experiments (Sun X et al. 2015).

### Reverse transcription-quantitative PCR (RT-qPCR)

TRIzol^®^ reagent (Invitrogen; Thermo Fisher Scientific, Inc., Waltham, MA) was used to extract total RNA from lung fibroblasts and pulmonary tissues, which was then reverse-transcribed to cDNA using superscript reverse transcriptase (Invitrogen; Thermo Fisher Scientific, Inc., Waltham, MA) with oligodeoxythymidine for mRNAs. A CFX Connect real-time PCR detection system (Bio-Rad Laboratories, Inc., Hercules, CA) was used to perform qPCR with the following primer sequences: Sirt3 (accession no. NM_001127351.1) forward, 5′-AGCAACCTTCAGCAGTATGACATCC-3′ and reverse, 5′-TTTCACAACGCCAGTACAGACAGG-3′; monocyte chemotactic protein-1 (MCP-1; accession no. NM_011333) forward,5′-CCACTCACCTGCTGCTACTCATTC-3′ and reverse, 5′-GTTCACTGTCACACTGGTCACTCC-3′; TNF-α (accession no. NM_001278601.1) forward, 5′-CACCACGCTCTTCTGTCTACTGAAC-3′ and reverse, 5′-TGACGGCAGAGAGGAGGTTGAC-3′; plasminogen activator inhibitor-1 (PAI-1; accession no. NM_008871.2) forward, 5′-TCAATGACTGGGTGGAAAGGCATAC-3′ and reverse, 5′-AGATGTTGGTGAGGGCGGAGAG-3′ MMP-10 (accession no. NM_019471.3) forward, 5′-GCCTACCAATCTGCTCAGCGTATC-3′ and reverse, 5′-TGAAGCCACCAACATCAGGAACAC-3′ reverse; and MMP-12 (accession no. NM_001320076.1) forward, 5′-TCAATGACTGGGTGGAAAGGCATAC-3′ and reverse, 5′-AGATGTTGGTGAGGGCGGAGAG-3′. The 20 μL reaction solution contained 5 μL diluted cDNA, 0.5 μM paired primer, 10 μL SYBRGreen mix (Aidlab Biotechnologies Co., Ltd., Beijing, China) and 4.9 μL DEPC water. The annealing temperature and amplification were set at 60 °C and 40 cycles, respectively. The comparative Cq method (2^–ΔΔCq^) was performed to measure the relative gene expression (Du JK et al. 2016). mRNA levels were normalized to those of β-actin.

### Western blotting

Cold RIPA lysis buffer (Beyotime Institute of Biotechnology, Shanghai, China) with Protease Inhibitor Cocktail (Roche Diagnostics, Basel, Switzerland) was used to lyse lung fibroblasts and pulmonary tissues. Following determination of protein concentrations using BCA assays, the isolated protein was separated using 10% SDS-PAGE and then transferred to PVDF membranes. Following blocking with non-fat dry milk dissolved in TBS for 2 h at room temperature, the membranes were incubated with BSA-diluted primary antibodies against Sirt3 (1:1000; cat. no. sc-365175; Santa Cruz Biotechnology, Inc., Dallas, TX), p16 (1:1000, cat. no. sc-56330; Santa Cruz Biotechnology, Inc., Dallas, TX), Smad4 (1:1000; cat. no. sc-7966; Santa Cruz Biotechnology, Inc., Dallas, TX), p-Smad2 (p-Smad2; 1:1000; Cell Signaling Technology, Inc., Danvers, MA), p-Smad3 (1:1000; Cell Signaling Technology, Inc., Danvers, MA), p53 (1:1000; cat. no. 10422-1-AP; ProteinTech Group, Inc., Rosemont, IL), p21 (1:1000; cat. no. 27296-1-AP; ProteinTech Group, Inc., Rosemont, IL), αSMA (1:1000, cat. no. sc-8432; Santa Cruz Biotechnology, Inc., Dallas, TX), fibronectin (1:1000, cat. no. sc-8422; Santa Cruz Biotechnology, Inc., Dallas, TX), Smad2 (1:1000; cat. no. sc-393312 Santa Cruz Biotechnology, Inc., Dallas, TX), Smad3 (1:1000; cat. no. sc-101154; Santa Cruz Biotechnology, Inc., Dallas, TX) or β-actin (1:1000; cat. no. sc-81178; Santa Cruz Biotechnology, Inc., Dallas, TX) at 4 °C overnight. Next, the membranes were incubated with a horseradish peroxidase-conjugated secondary antibody (1:1000) for 2 h at room temperature. An Enhanced Chemiluminescence Western Blotting Detection system (Santa Cruz Biotechnology, Inc., Dallas, TX) and a GeneGnome HR scanner (Syngene Europe, Frederick, MD) were used to visualize the bands.

### Statistical analysis

The data are presented as the mean ± SEM. Statistical analysis was performed using SPSS 16.0 (SPSS, Inc., Chicago, IL). Statistical comparisons between two groups were determined using a two-tailed Student’s *t*-test. One- or two-way ANOVA with a Bonferroni’s *post hoc* test was performed for comparisons among multiple groups. *p* < 0.05 was considered to indicate a statistically significant difference.

## Results

### Baicalein mitigates BLM-induced lung fibrosis in the mouse model

**Figure 1.**
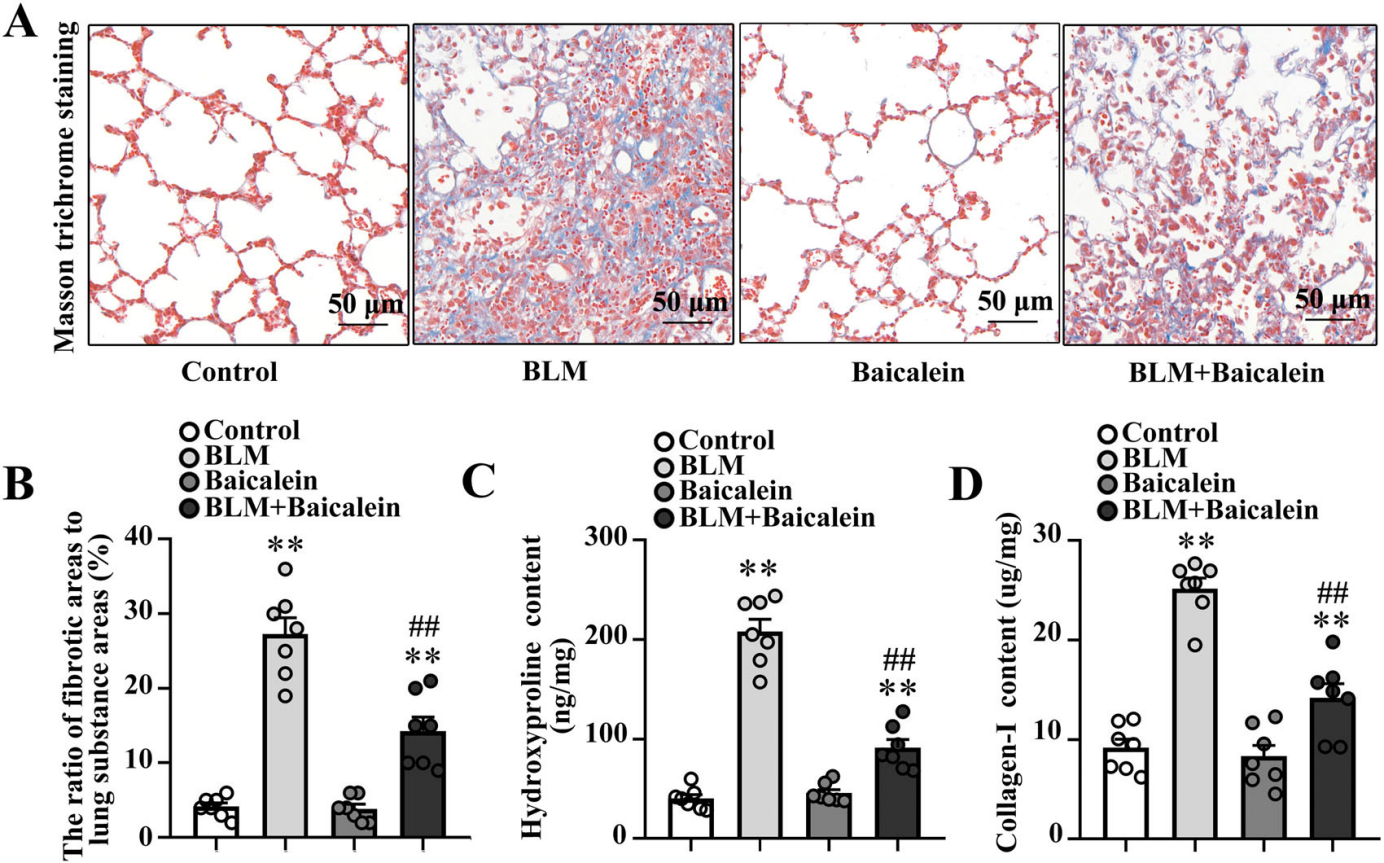
Baicalein mitigates BLM-induced lung fibrosis. (A, B) Masson’s trichrome staining was performed to measure collagen deposition in pulmonary tissues in control, BLM, baicalein and BLM + baicalein groups. Representative images (A) and changes in the ratio of collagen-deposited areas to lung substance areas (a morphometric measure of pulmonary fibrosis) (B). Hydroxyproline (C) and collagen I (D) contents in pulmonary tissues were examined by ELISA in control, BLM, baicalein and BLM + baicalein groups. Data are presented as the mean ± SEM (*n* = 7). **p < 0.01 vs. control. ^##^*p* *<* 0.01 vs. BLM. BLM: bleomycin.

The impact of baicalein on BLM-induced lung fibrosis was first explored. As demonstrated in Figure 1(A,B), Masson’s trichrome staining revealed considerable collagen deposition in the pulmonary interstitium, with collagen deposition primarily observed in the alveolar walls along with epithelial thickening and cellular infiltrates. BLM also increased the expression of fibronectin and α-SMA in the protein level of lung tissue (Fig. S1A). Baicalein could significantly mitigate BLM-induced collagen deposition, structural damage and elevated expression of fibronectin and α-SMA in the lung tissue, as evidenced by a decrease in the amount of collagen deposition, relatively normal pulmonary architecture and low expression of fibronectin and α-SMA (Figure 1(A,B), Fig. S1A).

Next, the amount of hydroxyproline and collagen I was measured to quantify the amount of collagen deposited in the lungs. As shown in Figure 1(C,D), the amount of hydroxyproline and collagen I was significantly increased in BLM-treated mice and baicalein could reverse the BLM-induced increase in the content of hydroxyproline and collagen I, indicating that baicalein mitigates BLM-induced lung fibrosis.

### Baicalein mitigates BLM-induced lung fibroblast senescence in the mouse model

**Figure 2.**
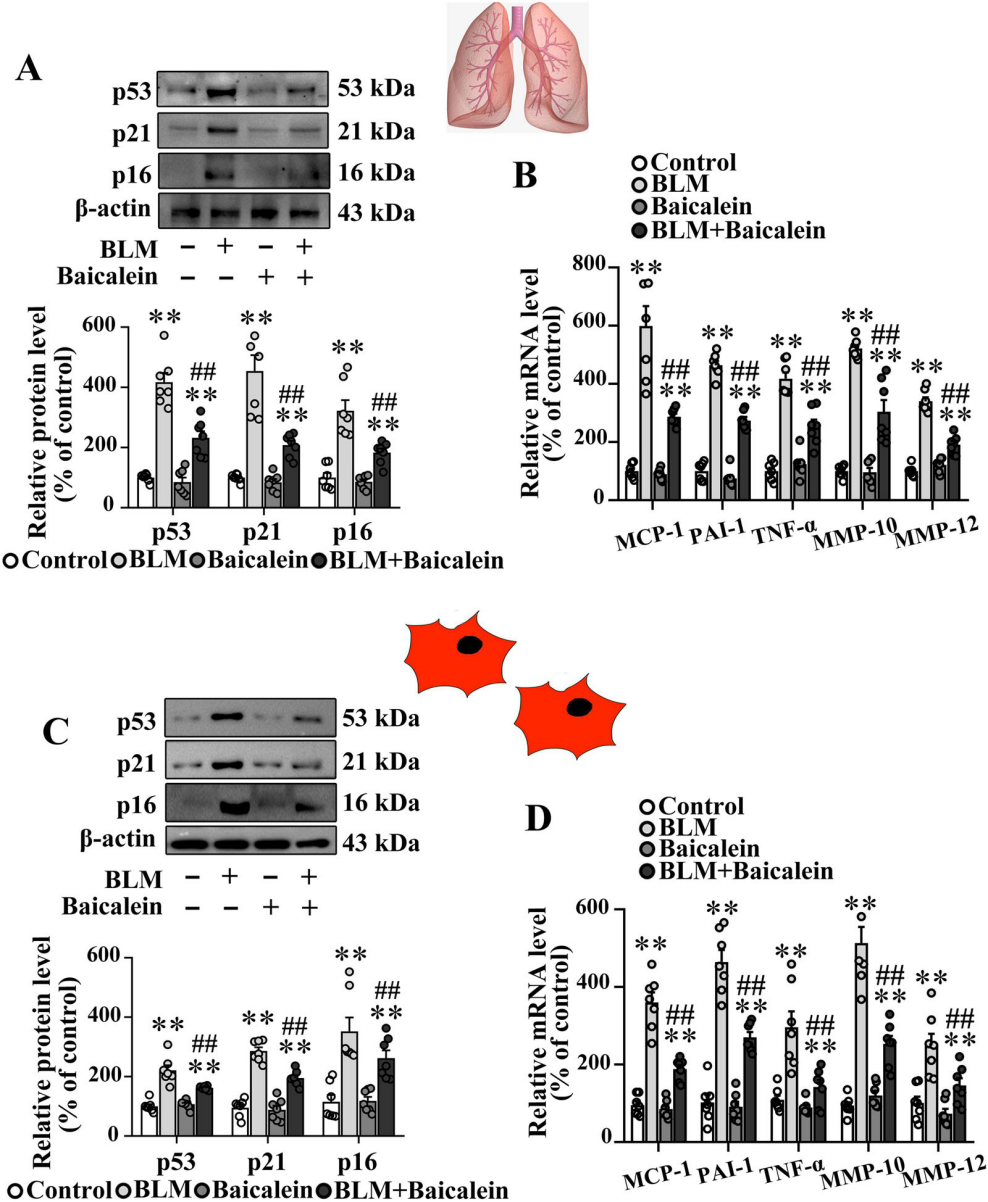
Baicalein mitigates BLM-induced senescence in lung tissues and isolated lung fibroblasts. (A, B) Protein levels of p53, p21 and p16 and the mRNA levels of SASP factors, including MCP-1, PAI-1, TNF-α, MMP-10 and MMP-12 in lung tissues were measured in control, BLM, baicalein and BLM + baicalein groups. (C, D) Protein levels of p53, p21 and p16 and the mRNA levels of MCP-1, PAI-1, TNF-α, MMP-10 and MMP-12 in isolated lung fibroblasts were measured using western blotting and RT-qPCR, respectively. Representative protein bands were presented on the top of the histograms (A, C). Data are presented as the mean ± SEM (*n* = 7). **p < 0.01 vs. control. ^##^*p* *<* 0.01 vs. BLM. SASP: senescence-associated secretory phenotype; MCP-1: monocyte chemotactic protein-1; PAI-1: plasminogen activator inhibitor-1; TNF-α: tumour necrosis factor-α; MMP: matrix metalloproteinase; RT-qPCR: reverse transcription-quantitative PCR; BLM: bleomycin.

Next, the effects of baicalein on BLM-induced cell senescence in the lung tissue were explored. As shown in Figure 2(A), the protein levels of senescence effectors, including p53, p21 and p16, were significantly elevated in the lung tissue of BLM-treated mice. The ratio of the SA-β-gal-positive senescent cells was also increased in isolated lung fibroblast of BLM-treated mice (Fig. S1B and C). However, the increased expression of p53, p21 and p16 and the elevated ratio of SA-β-gal-positive senescent cells were markedly reversed by baicalein treatment. In addition, the lung tissue in BLM-treated mice demonstrated markedly increased transcript levels of proinflammatory and profibrotic senescence-associated secretory phenotype (SASP) factors, including MCP-1, PAI-1, TNF-α, MMP-10 and MMP-12, which were partly attenuated by baicalein treatment (Figure 2(B)).

To further clarify the direct effect of baicalein on lung fibroblast senescence in BLM-treated mice, the lung fibroblasts in BLM-treated mice exhibited an obvious increase in the protein levels of p53, p21 and p16, as well as the transcript levels of proinflammatory and profibrotic SASP factors, and this was reversed by baicalein treatment (Figure 2(C,D)), indicating that baicalein mitigated BLM-induced fibroblast senescence.

### Baicalein ameliorates BLM-induced activation of TGF-β1/Smad signalling in lung tissues

**Figure 3.**
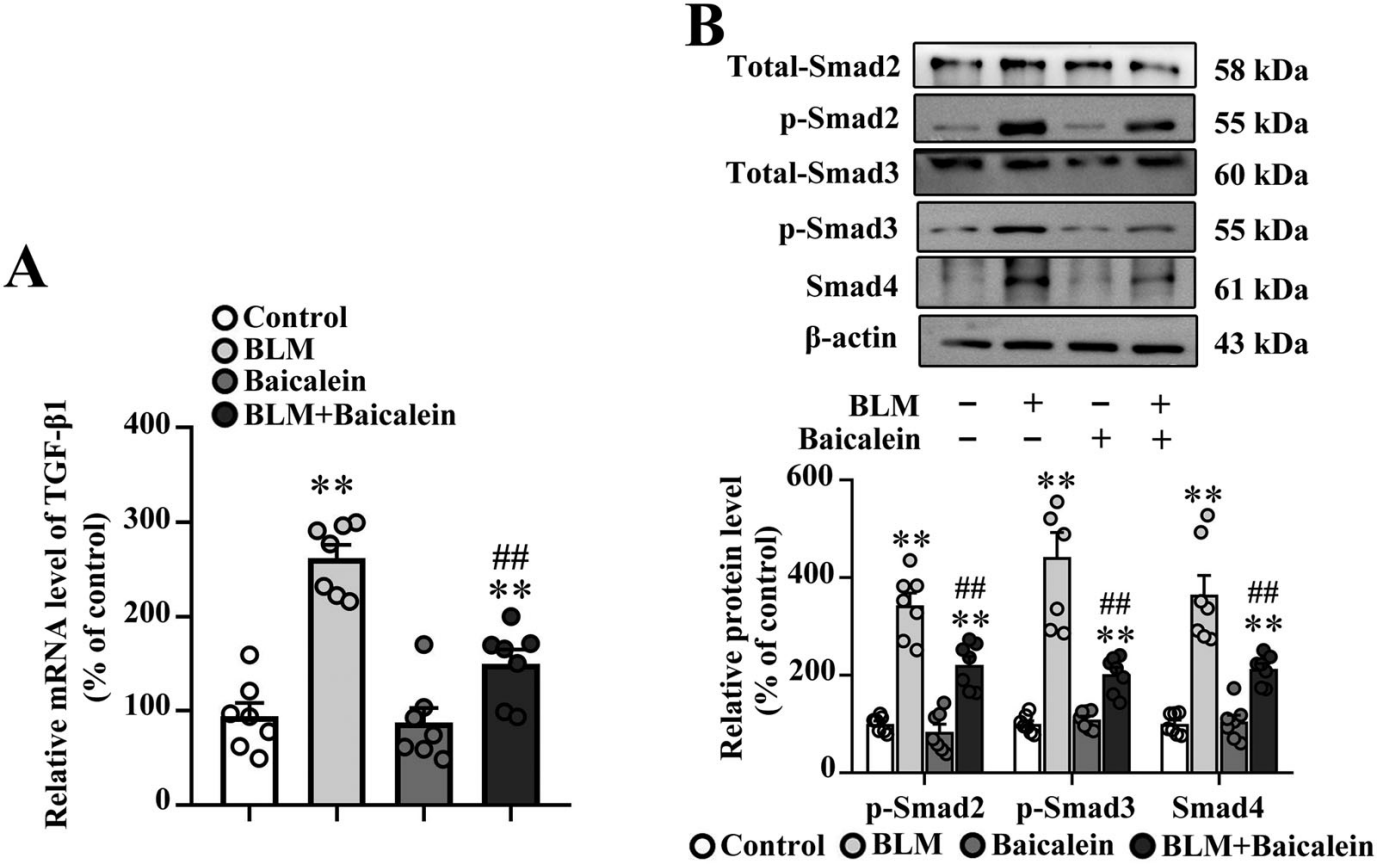
Baicalein mitigates BLM-induced TGF-β1/Smad signalling in lung tissue. (A) The mRNA expression levels of TGF-β1 in pulmonary tissues were examined using RT-qPCR. (B) The protein expression levels of p-Smad2, p-Smad3 and Smad4 in pulmonary tissues were examined using western blotting. Representative immunoblots and the corresponding histograms are presented. Data are presented as the mean ± SEM (*n* = 7). **p < 0.01 vs. control. ^##^*p* *<* 0.01 vs. BLM. BLM: bleomycin; TGF-β1: transforming growth factor-β1; Smad: mothers against decapentaplegic homolog; RT-qPCR: reverse transcription-quantitative PCR.

The TGF-β1/Smad pathway has been widely reported to play a crucial role in tissue fibrosis (Tseliou et al. 2014; Tran et al. 2019; Yao et al. 2019; Chale-Dzul et al. 2020; Hussein et al. 2020; Li X-F et al. 2020; Du J-K et al. 2021). As shown in Figure 3(A,B), the transcript levels of TGF-β1 and protein levels of p-Smad2, p-Smad3 and Smad4 were increased in the lung tissue of BLM-treated mice compared with the control, suggesting that BLM activates TGF-β1-Smad signalling in the lung. It was also found that baicalein could suppress the BLM-induced TGF-β1 production, p-Smad2, p-Smad3 and Smad4 expression in the lung.

### Baicalein restores Sirt3 expression in the lung tissues of BLM-treated mice

**Figure 4.**
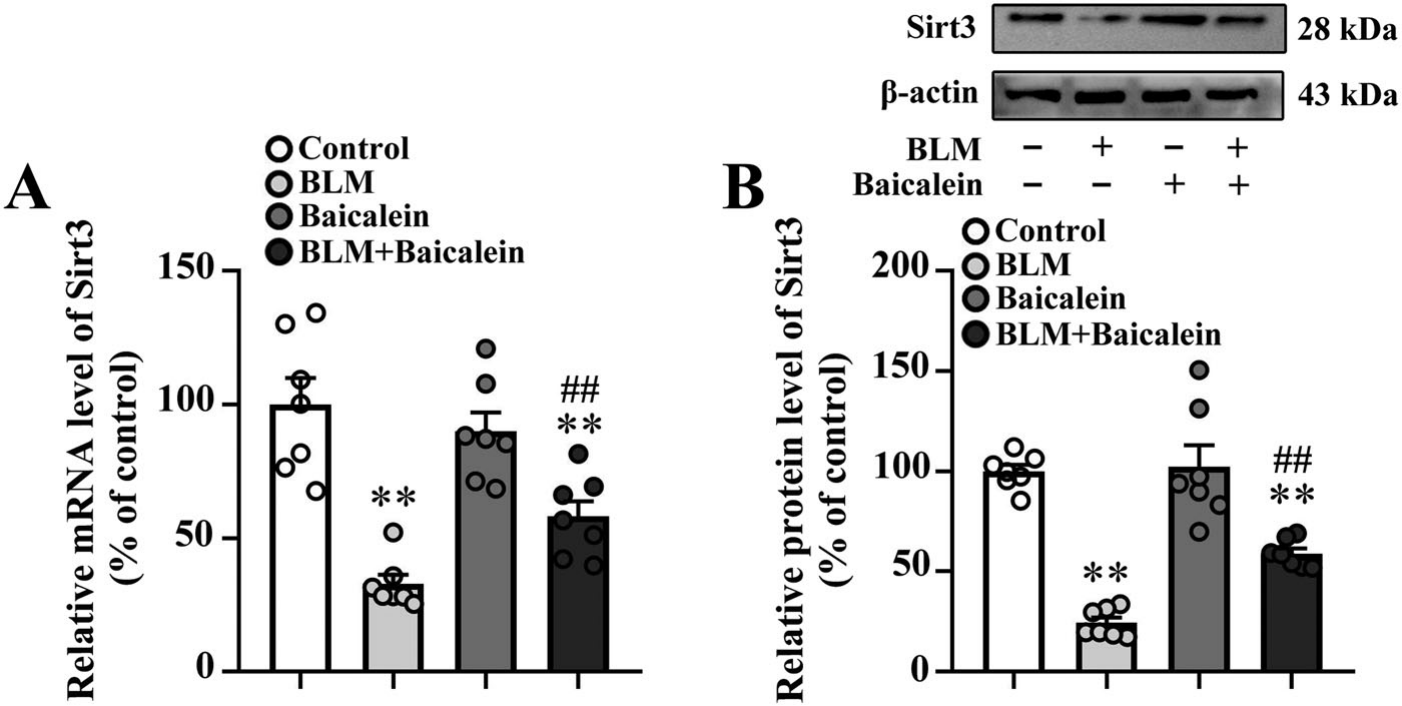
Baicalein prevents BLM-induced downregulation of Sirt3 in lung tissues. RT-qPCR and western blotting analysis were performed to measure the (A) mRNA and (B) protein Sirt3 expression and in the lung tissues of control, BLM, baicalein and BLM + baicalein groups. Representative immunoblots of Sirt3 and the corresponding histograms are presented. Data are presented as the mean ± SEM (*n* = 7). **p < 0.01 vs. control. ^##^*p* < 0.01 vs. BLM. p-: phosphorylated. Sirt3: sirtuin 3; BLM: bleomycin.

A previous study indicated that the downregulation of Sirt3 contributes to aging-associated tissue fibrosis by blocking TGF-β expression (Sundaresan et al. 2015). As shown in Figure 4(A,B), BLM treatment resulted in an obvious decrease in the mRNA and protein expression levels of Sirt3 in the lungs. Baicalein could prevent the BLM-induced decrease in the lung tissue expression of Sirt3.

### Sirt3 siRNA abrogates the protective role of baicalein against BLM-induced pulmonary fibrosis

**Figure 5.**
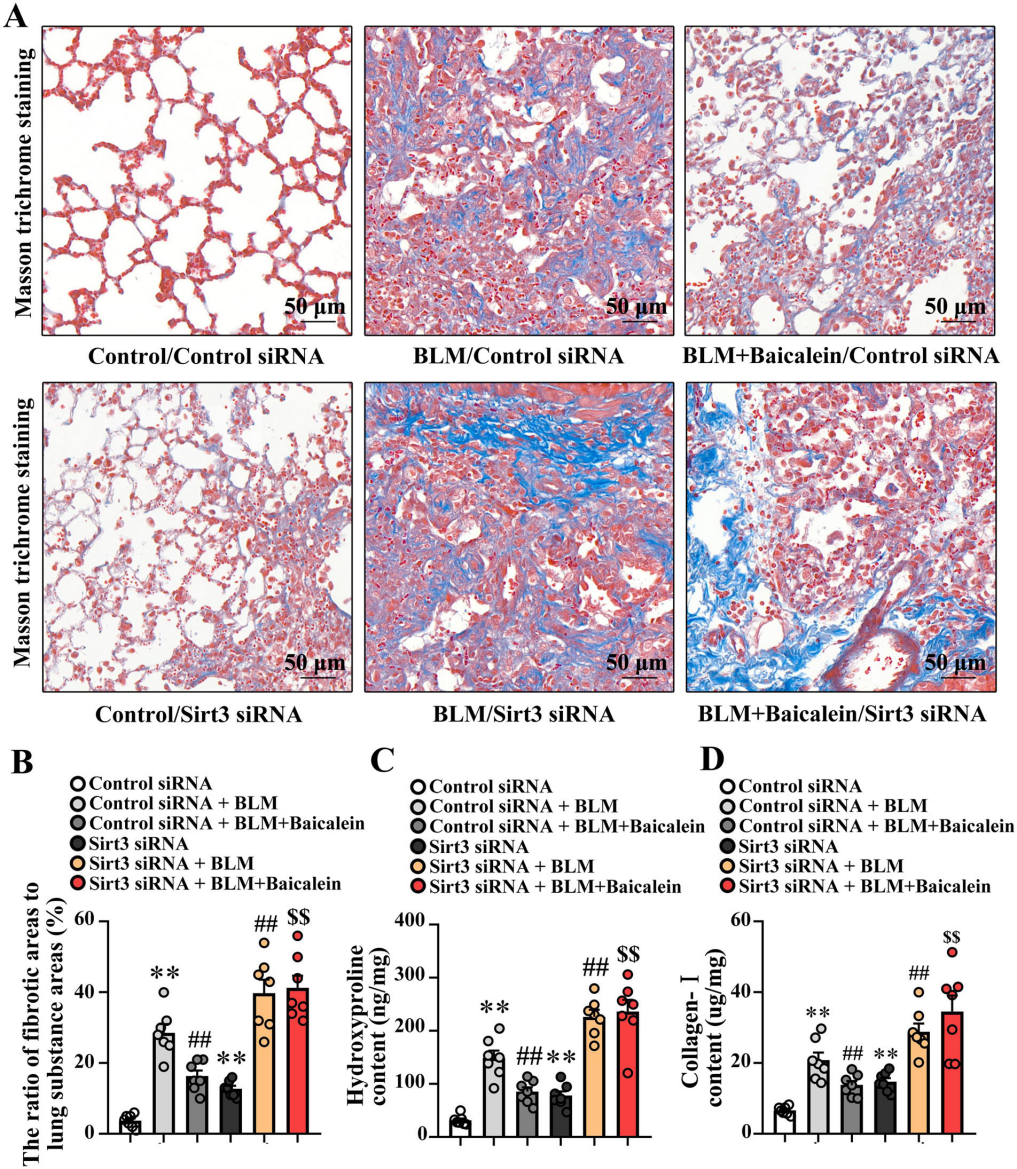
Silencing of Sirt3 abolishes the protective effect of baicalein against BLM-induced lung fibrosis. Masson’s trichrome staining was performed to measure collagen deposition in pulmonary tissues. (A, B) Representative images and the ratio of fibrotic areas to the total lung area. (C) Hydroxyproline and (D) collagen I content in pulmonary tissues was examined using ELISA. Data are presented as the mean ± SEM (*n* = 7). **p < 0.01 vs. control. ^##^*p* *<* 0.01 vs. BLM. ^$$^*p* < 0.01 vs. BLM + baicalein. Sirt3: sirtuin 3; BLM: bleomycin.

Next, the impact of Sirt3 siRNA on the protective role of baicalein against BLM-induced pulmonary fibrosis was explored. As shown in Figure 5(A), Sirt3 siRNA resulted in an ∼80% decrease in Sirt3 expression in the lung tissues, and Sirt3 knockdown blocked the protective effects of baicalein against BLM-induced pulmonary fibrosis, as evidenced by the decreased collagen deposition (Figure 5(B)), and the levels of hydroxyproline (Figure 5(C)), collagen I (Figure 5(D)), aSMA and fibronectin (Fig. S2A and B). These results suggested that Sirt3 siRNA could abolish the protective role of baicalein against BLM-induced pulmonary fibrosis.

### Sirt3 siRNA abolishes the protective role of baicalein against BLM-induced lung fibroblast senescence and activation of TGF-β1/Smad signalling in lung tissues

**Figure 6.**
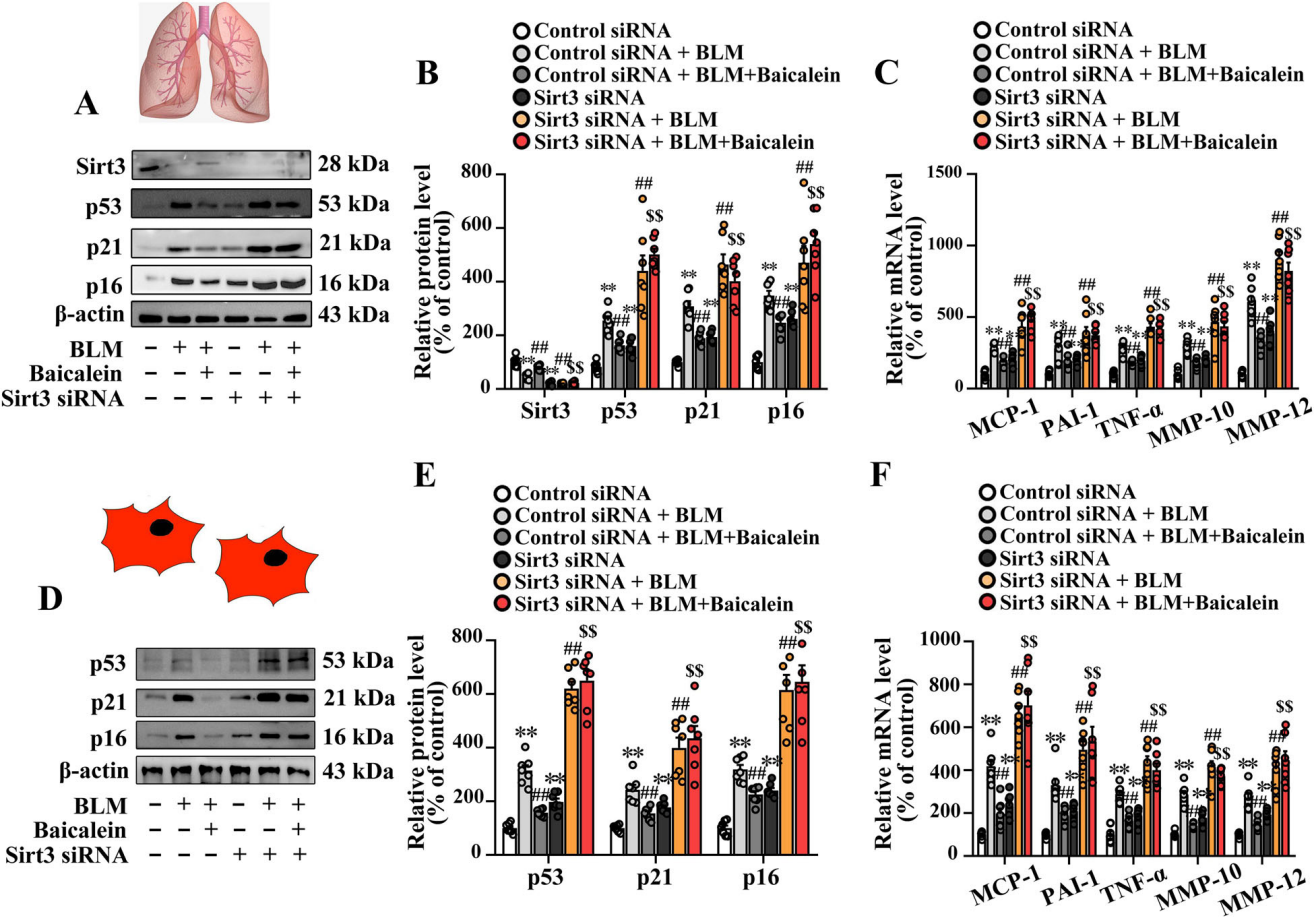
Sirt3 silencing abolishes the protective effect of baicalein against BLM-induced senescence in lung tissue and isolated lung fibroblasts. (A, B, D and E) The protein expression levels of Sirt3, p53, p21 and p16 in pulmonary tissues were measured using western blotting. (C, F) The mRNA levels of MCP-1, PAI-1, TNF-α, MMP-10 and MMP-12 were measured using RT-qPCR. Data are presented as the mean ± SEM (*n* = 7). **p < 0.01 vs. control. ^##^*p* *<* 0.01 vs. BLM. ^$$^*p* < 0.01 vs. BLM + baicalein. Sirt3: sirtuin 3; BLM: bleomycin; MCP-1: monocyte chemotactic protein-1; PAI-1: plasminogen activator inhibitor-1; TNF-α: tumour necrosis factor-α; MMP: matrix metalloproteinase; RT-qPCR: reverse transcription-quantitative PCR.

Next, the impact of Sirt3 siRNA on the beneficial role of baicalein against BLM-induced lung fibroblast senescence was explored. As shown in Figure 6, Fig. S2C and D, Sirt3 siRNA blocked the protective effects of baicalein against BLM-induced senescence and the alteration of the levels of proinflammatory and profibrotic SASP factors in the lung tissue and isolated lung fibroblasts, as evidenced by the decreased protein levels of p53, p21, p16 and the ratio of the SA-β-gal-positive senescent cells in isolated lung fibroblast, as well as the transcript levels of proinflammatory and profibrotic SASP factors.

**Figure 7.**
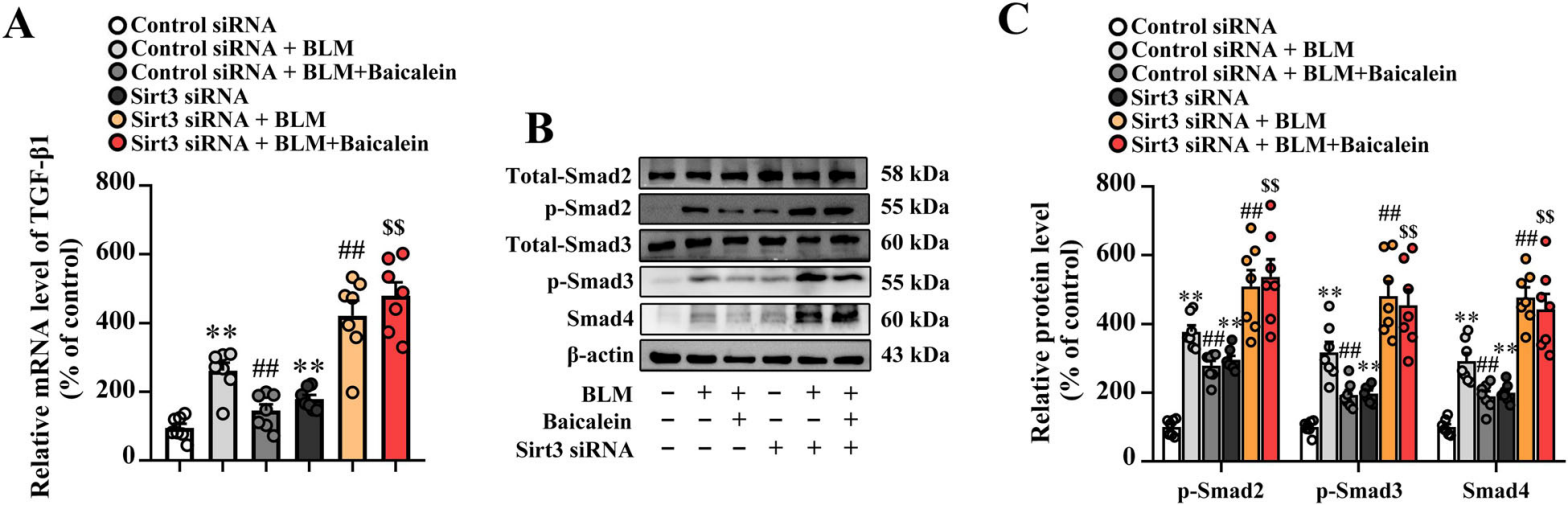
Sirt3 knockdown abolishes the protective effects of baicalein on BLM-induced TGF-β1/Smad signalling in the lung tissue. (A) The mRNA expression levels of TGF-β1 in pulmonary tissues were examined using RT-qPCR. (B) The protein expression levels of p-Smad2, p-Smad3 and Smad4 in pulmonary tissues were examined using western blotting. Data are presented as the mean ± SEM (*n* = 7). **p < 0.01 vs. control. ^##^*p* *<* 0.01 vs. BLM. ^$$^*p* < 0.01 vs. BLM + baicalein. Sirt3: sirtuin 3; BLM: bleomycin; TGF-β1: transforming growth factor-β1; Smad: mothers against decapentaplegic homolog; RT-qPCR: reverse transcription-quantitative PCR.

We then explored the impact of Sirt3 siRNA on the role of baicalein against BLM-induced activation of TGF-β1-smad pathway. Results showed that Sirt3 siRNA blocked the inhibitory effect of baicalein against BLM-induced activation of TGF-β1/Smad signalling, as shown by increased transcript levels of TGF-β1 (Figure 7(A)) and protein levels of p-Smad2, p-Smad3 and Smad4 (Figure 7(B,C)) in the lung tissue.

## Discussion

IPF is a chronic, progressive, fibrosing interstitial lung disease that affects hundreds of thousands of people worldwide, reducing their quality of life and leading to death from respiratory failure within years of diagnosis. Herein, BLM administration was found to result in significant pulmonary fibrosis and inflammation, and baicalein was found to play a positive role in preventing BLM-induced fibrosis and inflammatory responses in a mouse model. Our findings suggested that the dysregulation of Sirt3-mediated pulmonary fibroblast senescence contributed to BLM-induced lung fibrosis, and Sirt3 knockdown blocked the protective role of baicalein in preventing BLM-induced fibrosis.

Baicalein is a common plant flavonoid with a wide range of beneficial pharmacological properties, including anti-inflammatory (Teng et al. 2020; D’Amico et al. 2021), antioxidative (D'Amico et al. 2019), anti-apoptotic (Liu C et al. 2010; Li X-x et al. 2012; Lin M et al. 2014; Hung et al. 2016; Wang M et al. 2020), antitumorigenic (Li J et al. 2019) and pro-immunoregulatory functions (Shi et al. 2018). In addition, previous studies suggested that baicalein has antifibrotic potential in several types of tissues, including the lung (Gao et al. 2013), kidney (Hu et al. 2017), liver (Sun H et al. 2010) and heart (Kong et al. 2010). Baicalein has been reported to reverse BLM-induced lung fibrosis in rats, which was partly achieved through the inhibition of TGF-β/Smad signalling. Consistent with these findings, it was also found that baicalein could attenuate BLM-induced pulmonary fibrosis in mice. The protective role of baicalein against BLM-induced pulmonary fibrosis was partly independent in inhibiting TGF-β/Smad signalling, as shown by the improved lung architecture, decreased collagen deposition and amounts of hydroxyproline and type I collagen, as well as TGF-β1 production and phosphorylation of Smad2, Smad3 and Smad4 expression in the lung.

Cell senescence refers to a relatively stable state in which cells irreversibly leave the cell cycle and lose their proliferative ability under the action of signal transduction. In recent decades, cell senescence has attracted widespread attention as it increases the morbidity of fibroproliferative pulmonary diseases in elderly individuals (Parimon et al. 2021). Recent studies have demonstrated that epithelial progenitor cell dysfunction and cellular senescence, including epithelial and fibroblast senescence, were associated with the pathological development of IPF (Demaria et al. 2014; Lehmann et al. 2017). Hohmann et al. (2019) found that quercetin could attenuate BLM-induced lung fibrosis and injury by inhibiting fibroblast senescence and enhancing FasL- or TRAIL-induced apoptosis. Of note, Cui et al. (2018) demonstrated that baicalein could mitigate TGF-β1-mediated lung FMT differentiation through the inhibition of miR-21 expression. In the present study, it was found that baicalein reversed BLM-induced lung fibroblast senescence and increased the transcript levels of proinflammatory and profibrotic SASP factors in mice. However, whether baicalein enhances lung fibroblast apoptosis in BLM-treated mice and regulates the expression of miR-21 is worthy of further study.

Sirt3 dysregulation was reported to be involved in the pathological process of lung fibrosis. Clinically, it was demonstrated that Sirt3 was absent within fibrotic areas, as compared with adjacent areas within the same tissue in the scleroderma and IPF specimens (Sosulski et al. 2017). It was also reported that Sirt3 was deficient in the alveolar epithelial cells of IPF patients (Cheresh et al. 2021); these findings indicated that Sirt3 may have therapeutic potential in the management of lung fibrosis. In animal models of pulmonary fibrosis, the expression of Sirt3 was significantly decreased in the lung tissue of Ad-TGF-β1-treated mice (Sosulski et al. 2017). Sirt3 overexpression attenuated asbestos-mediated lung fibrosis and the beneficial effect of Sirt3 overexpression was associated with decreased lung mtDNA damage and Mo-AM recruitment (Jacobs et al. 2008). Consistent with the study, Sosulski et al. (2017) found that Sirt3-deficient mice were susceptible to lung fibrosis, and Sirt3 overexpression mitigated TGF-β1-induced FMT differentiation. The role of Sirt3 in lung fibrosis and FMT differentiation was associated with Smad3 (Schafer et al. 2017). Nevertheless, it was also found that Sirt3 expression was significantly decreased in the lung tissues of BLM-treated mice and Sirt3 downregulation contributed to the pathological process of BLM-mediated pulmonary fibrosis.

Located in the mitochondrial matrix, Sirt3 is a member of the Sirtuin family. It was reported to deacetylate and activate mitochondrial forkhead box class O 3a, which then regulated defective mitochondrial clearance through transcriptional regulation of autophagy-related genes (Jacobs et al. 2008). In addition, Im et al. (2015) showed that autophagy was deficient in lung fibroblasts and in the fibrotic lungs of IPF patients. A limitation of the present study was that it did not examine mitochondrial function and autophagy. In addition, no *in vitro* experiments were performed in the present study, which would have improved its relevance for IPF and/or senescence. Inhibition experiments with baicalein using primary human IPF fibroblasts should be performed, and the robustness of senescence markers can be improved by performing senescence-associated-β-galactosidase staining and measuring SASP factors, in addition to gene expression, using ELISA.

## Conclusions

**Figure 8.**
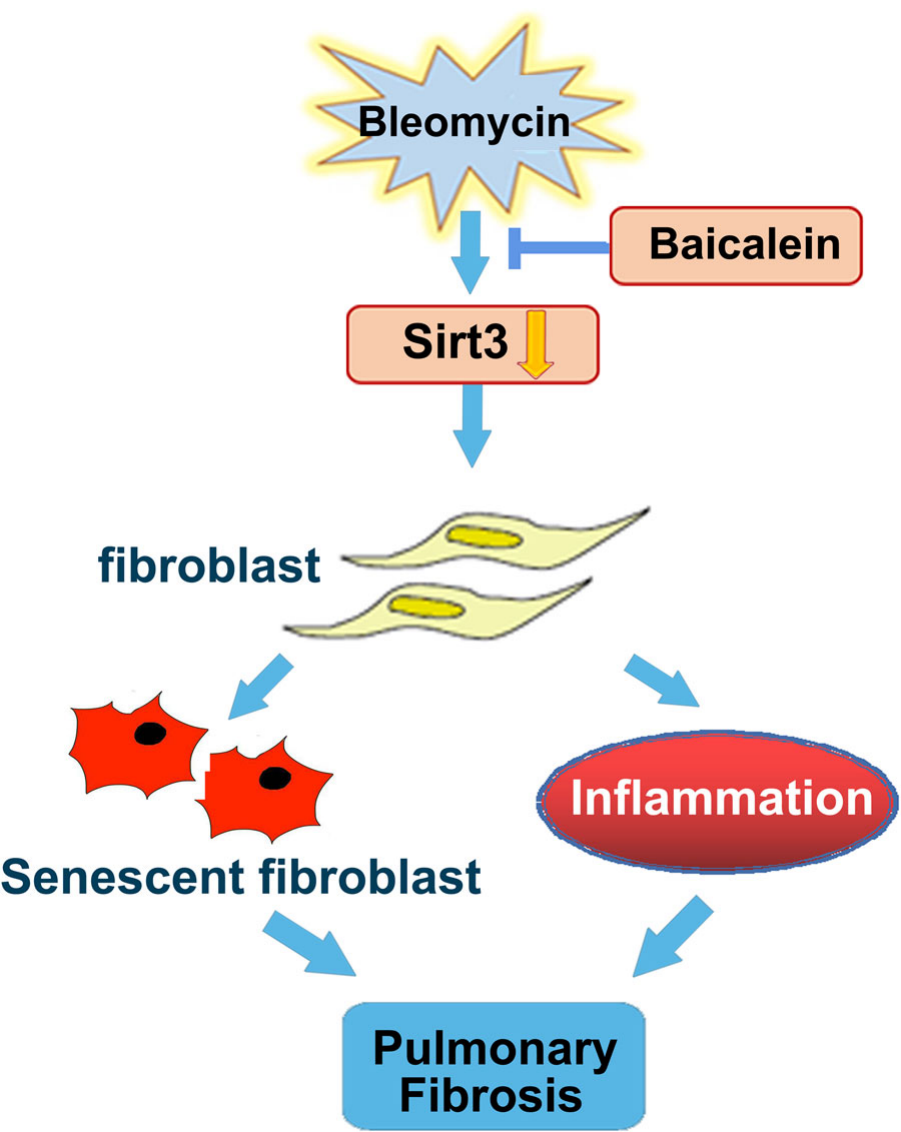
Schematic diagram of the mechanism through which baicalein attenuates BLM-induced pulmonary fibrosis through upregulating Sirt3 expression. BLM suppressed Sirt3 expression, and Sirt3 dysregulation contributed to pulmonary fibroblast senescence and inflammation, resulting in pulmonary fibrosis, which was markedly mitigated by baicalein administration. BLM: bleomycin; Sirt3: sirtuin 3.

Baicalein inhibited the BLM-mediated activation of TGF-β1/Smad and lung fibroblast senescence, which was in parallel with the protective roles of baicalein against BLM-mediated lung fibrosis. Furthermore, baicalein preserved the BLM-induced downregulation of lung Sirt3 expression, and thus the suppression of TGF-β1/Smad signalling pathway and lung fibrosis, which might provide an experimental basis for treatment of IPF (Figure 8).

## Supplementary Material

Supplemental Material

Supplemental Material

## References

[CIT0001] Alvarez D, Cardenes N, Sellares J, Bueno M, Corey C, Hanumanthu VS, Peng Y, D'Cunha H, Sembrat J, Nouraie M, et al. 2017. IPF lung fibroblasts have a senescent phenotype. Am J Physiol Lung Cell Mol Physiol. 313(6):L1164–L1173. 28860144 10.1152/ajplung.00220.2017 PMC6148001

[CIT0002] Blokland KEC, Habibie H, Borghuis T, Teitsma GJ, Schuliga M, Melgert BN, Knight DA, Brandsma CA, Pouwels SD, Burgess JK. 2021. Regulation of cellular senescence is independent from profibrotic fibroblast-deposited ECM. Cells. 10(7):1628. 34209854 10.3390/cells10071628 PMC8307656

[CIT0003] Cao X, Li Y, Shi J, Tang H. 2021. The potentially therapeutic role of EPAC in curbing the process of idiopathic pulmonary fibrosis via differential cellular pathways. J Inflamm Res. 14:611–619. 33679138 10.2147/JIR.S296382 PMC7926039

[CIT0004] Chale-Dzul J, Perez-Cabeza de Vaca R, Quintal-Novelo C, Olivera-Castillo L, Moo-Puc R. 2020. Hepatoprotective effect of a fucoidan extract from *Sargassum fluitans* Borgesen against CCl_4_-induced toxicity in rats. Int J Biol Macromol. 145:500–509. 31874267 10.1016/j.ijbiomac.2019.12.183

[CIT0005] Chen T, Guo Y, Wang J, Ai L, Ma L, He W, Li Z, Yu X, Li J, Fan X, et al. 2021. LncRNA CTD-2528L19.6 prevents the progression of IPF by alleviating fibroblast activation. Cell Death Dis. 12(6):600. 34112765 10.1038/s41419-021-03884-5 PMC8192779

[CIT0006] Chen Y, Zhao Z, Li Y, Yang Y, Li L, Jiang Y, Lin C, Cao Y, Zhou P, Tian Y, et al. 2021. Baicalein alleviates hyperuricemia by promoting uric acid excretion and inhibiting xanthine oxidase. Phytomedicine. 80:153374. 33075645 10.1016/j.phymed.2020.153374

[CIT0007] Cheresh P, Kim S-J, Jablonski R, Watanabe S, Lu Z, Chi M, Helmin KA, Gius D, Budinger GRS, Kamp DW. 2021. SIRT3 overexpression ameliorates asbestos-induced pulmonary fibrosis, mt-DNA damage, and lung fibrogenic monocyte recruitment. Int J Mol Sci. 22(13):6856. 34202229 10.3390/ijms22136856 PMC8268084

[CIT0008] Cui X, Sun X, Lu F, Jiang X. 2018. Baicalein represses TGF-beta1-induced fibroblast differentiation through the inhibition of miR-21. Toxicol Appl Pharmacol. 358:35–42. 30201452 10.1016/j.taap.2018.09.007

[CIT0009] D'Amico R, Fusco R, Gugliandolo E, Cordaro M, Siracusa R, Impellizzeri D, Peritore AF, Crupi R, Cuzzocrea S, Di Paola R. 2019. Effects of a new compound containing palmitoylethanolamide and baicalein in myocardial ischaemia/reperfusion injury *in vivo*. Phytomedicine. 54:27–42. 30668378 10.1016/j.phymed.2018.09.191

[CIT0010] D’Amico R, Genovese T, Cordaro M, Siracusa R, Gugliandolo E, Peritore AF, Interdonato L, Crupi R, Cuzzocrea S, Di Paola R, et al. 2021. Palmitoylethanolamide/baicalein regulates the androgen receptor signaling and NF-kappaB/Nrf2 pathways in benign prostatic hyperplasia. Antioxidants. 10(7):1014. 34202665 10.3390/antiox10071014 PMC8300753

[CIT0011] Demaria M, Ohtani N, Youssef SA, Rodier F, Toussaint W, Mitchell JR, Laberge RM, Vijg J, Van Steeg H, Dolle ME, et al. 2014. An essential role for senescent cells in optimal wound healing through secretion of PDGF-AA. Dev Cell. 31(6):722–733. 25499914 10.1016/j.devcel.2014.11.012 PMC4349629

[CIT0012] Du JK, Cong BH, Yu Q, Wang H, Wang L, Wang CN, Tang XL, Lu JQ, Zhu XY, Ni X. 2016. Upregulation of microRNA-22 contributes to myocardial ischemia–reperfusion injury by interfering with the mitochondrial function. Free Radic Biol Med. 96:406–417. 27174562 10.1016/j.freeradbiomed.2016.05.006

[CIT0013] Du J-K, Yu Q, Liu Y-J, Du S-F, Huang L-Y, Xu D-H, Ni X, Zhu X-Y. 2021. A novel role of kallikrein-related peptidase 8 in the pathogenesis of diabetic cardiac fibrosis. Theranostics. 11(9):4207–4231. 33754057 10.7150/thno.48530 PMC7977470

[CIT0014] Du S-F, Wang X-L, Ye C-L, He Z-J, Li D-X, Du B-R, Liu Y-J, Zhu X-Y. 2019. Exercise training ameliorates bleomycin-induced epithelial mesenchymal transition and lung fibrosis through restoration of H_2_S synthesis. Acta Physiol. 225(2):e13177. 10.1111/apha.13177 30136377

[CIT0015] Gao Y, Lu J, Zhang Y, Chen Y, Gu Z, Jiang X. 2013. Baicalein attenuates bleomycin-induced pulmonary fibrosis in rats through inhibition of miR-21. Pulm Pharmacol Ther. 26(6):649–654. 23523661 10.1016/j.pupt.2013.03.006

[CIT0016] Hecker L, Logsdon NJ, Kurundkar D, Kurundkar A, Bernard K, Hock T, Meldrum E, Sanders YY, Thannickal VJ. 2014. Reversal of persistent fibrosis in aging by targeting Nox4–Nrf2 redox imbalance. Sci Transl Med. 6:231–247. 10.1126/scitranslmed.3008182 PMC4545252 24718857

[CIT0017] Hohmann MS, Habiel DM, Coelho AL, Verri WA Jr, Hogaboam CM. 2019. Quercetin enhances ligand-induced apoptosis in senescent idiopathic pulmonary fibrosis fibroblasts and reduces lung fibrosis *in vivo*. Am J Respir Cell Mol Biol. 60(1):28–40. 30109946 10.1165/rcmb.2017-0289OC PMC6348716

[CIT0018] Hu Q, Gao L, Peng B, Liu X. 2017. Baicalin and baicalein attenuate renal fibrosis *in vitro* via inhibition of the TGF-β1 signaling pathway. Exp Ther Med. 14(4):3074–3080. 28928802 10.3892/etm.2017.4888 PMC5590043

[CIT0019] Hung KC, Huang HJ, Wang YT, Lin AM. 2016. Baicalein attenuates α-synuclein aggregation, inflammasome activation and autophagy in the MPP^+^-treated nigrostriatal dopaminergic system *in vivo*. J Ethnopharmacol. 194:522–529. 27742410 10.1016/j.jep.2016.10.040

[CIT0020] Hussein KH, Park KM, Yu L, Kwak HH, Woo HM. 2020. Decellularized hepatic extracellular matrix hydrogel attenuates hepatic stellate cell activation and liver fibrosis. Mater Sci Eng C Mater Biol Appl. 116:111160. 32806289 10.1016/j.msec.2020.111160

[CIT0021] Im J, Hergert P, Nho RS. 2015. Reduced FoxO3a expression causes low autophagy in idiopathic pulmonary fibrosis fibroblasts on collagen matrices. Am J Physiol Lung Cell Mol Physiol. 309(6):L552–L561. 26186945 10.1152/ajplung.00079.2015 PMC4572418

[CIT0022] Jacobs KM, Pennington JD, Bisht KS, Aykin-Burns N, Kim H-S, Mishra M, Sun L, Nguyen P, Ahn B-H, Leclerc J, et al. 2008. SIRT3 interacts with the daf-16 homolog FOXO3a in the mitochondria, as well as increases FOXO3a dependent gene expression. Int J Biol Sci. 4(5):291–299. 18781224 10.7150/ijbs.4.291 PMC2532794

[CIT0023] Kong EKC, Huang Y, Sanderson JE, Chan K-B, Yu S, Yu C-M. 2010. Baicalein and wogonin inhibit collagen deposition in SHR and WKY cardiac fibroblast cultures. BMB Rep. 43(4):297–303. 20423617 10.5483/bmbrep.2010.43.4.297

[CIT0024] Lehmann M, Korfei M, Mutze K, Klee S, Skronska-Wasek W, Alsafadi HN, Ota C, Costa R, Schiller HB, Lindner M, et al. 2017. Senolytic drugs target alveolar epithelial cell function and attenuate experimental lung fibrosis *ex vivo*. Eur Respir J. 50(2):1602367. 28775044 10.1183/13993003.02367-2016 PMC5593348

[CIT0025] Li J, Tian C, Xia Y, Mutanda I, Wang K, Wang Y. 2019. Production of plant-specific flavones baicalein and scutellarein in an engineered *E. coli* from available phenylalanine and tyrosine. Metab Eng. 52:124–133. 30496827 10.1016/j.ymben.2018.11.008

[CIT0026] Li X-F, Zhang S-H, Liu G-F, Yu S-N. 2020. miR-363 alleviates detrusor fibrosis via the TGF-beta1/Smad signaling pathway by targeting Col1a2 in rat models of STZ-induced T2DM. Mol Ther Nucleic Acids. 22:1142–1153. 33294298 10.1016/j.omtn.2020.07.001 PMC7695978

[CIT0027] Li X-x, He G-r, Mu X, Xu B, Tian S, Yu X, Meng F-r, Xuan Z-h, Du G-h 2012. Protective effects of baicalein against rotenone-induced neurotoxicity in PC12 cells and isolated rat brain mitochondria. Eur J Pharmacol. 674(2–3):227–233. 21996316 10.1016/j.ejphar.2011.09.181

[CIT0028] Li Y, Zhao J, Holscher C. 2017. Therapeutic potential of baicalein in Alzheimer’s disease and Parkinson’s disease. CNS Drugs. 31(8):639–652. 28634902 10.1007/s40263-017-0451-y

[CIT0029] Lin M, Li L, Li L, Pokhrel G, Qi G, Rong R, Zhu T. 2014. The protective effect of baicalin against renal ischemia–reperfusion injury through inhibition of inflammation and apoptosis. BMC Complement Altern Med. 14:19. 24417870 10.1186/1472-6882-14-19 PMC3893527

[CIT0030] Liu A, Wang W, Fang H, Yang Y, Jiang X, Liu S, Hu J, Hu Q, Dahmen U, Dirsch O. 2015. Baicalein protects against polymicrobial sepsis-induced liver injury via inhibition of inflammation and apoptosis in mice. Eur J Pharmacol. 748:45–53. 25533331 10.1016/j.ejphar.2014.12.014

[CIT0031] Liu C, Wu J, Xu K, Cai F, Gu J, Ma L, Chen J. 2010. Neuroprotection by baicalein in ischemic brain injury involves PTEN/AKT pathway. J Neurochem. 112(6):1500–1512. 20050973 10.1111/j.1471-4159.2009.06561.x

[CIT0032] Ma H, Liu S, Li S, Xia Y. 2022. Targeting growth factor and cytokine pathways to treat idiopathic pulmonary fibrosis. Front Pharmacol. 13:918771. 35721111 10.3389/fphar.2022.918771 PMC9204157

[CIT0033] Parimon T, Hohmann MS, Yao C. 2021. Cellular senescence: pathogenic mechanisms in lung fibrosis. Int J Mol Sci. 22(12):6214. 34207528 10.3390/ijms22126214 PMC8227105

[CIT0035] Raghu G, Remy-Jardin M, Richeldi L, Thomson CC, Inoue Y, Johkoh T, Kreuter M, Lynch DA, Maher TM, Martinez FJ, et al. 2022. Idiopathic pulmonary fibrosis (an update) and progressive pulmonary fibrosis in adults: an Official ATS/ERS/JRS/ALAT Clinical Practice Guideline. Am J Respir Crit Care Med. 205(9):e18–e47. 35486072 10.1164/rccm.202202-0399ST PMC9851481

[CIT0036] Röhrich M, Leitz D, Glatting FM, Wefers AK, Weinheimer O, Flechsig P, Kahn N, Mall MA, Giesel FL, Kratochwil C, et al. 2022. Fibroblast activation protein-specific PET/CT imaging in fibrotic interstitial lung diseases and lung cancer: a translational exploratory study. J Nucl Med. 63(1):127–133. 34272325 10.2967/jnumed.121.261925 PMC8717194

[CIT0037] Rui W, Li S, Xiao H, Xiao M, Shi J. 2020. Baicalein attenuates neuroinflammation by inhibiting NLRP3/caspase-1/GSDMD pathway in MPTP induced mice model of Parkinson’s disease. Int J Neuropsychopharmacol. 23(11):762–773. 32761175 10.1093/ijnp/pyaa060 PMC7745250

[CIT0038] Schafer MJ, White TA, Iijima K, Haak AJ, Ligresti G, Atkinson EJ, Oberg AL, Birch J, Salmonowicz H, Zhu Y, et al. 2017. Cellular senescence mediates fibrotic pulmonary disease. Nat Commun. 8:14532. 28230051 10.1038/ncomms14532 PMC5331226

[CIT0039] Shi R, Zhu D, Wei Z, Fu N, Wang C, Liu L, Zhang H, Liang Y, Xing J, Wang X, et al. 2018. Baicalein attenuates monocrotaline-induced pulmonary arterial hypertension by inhibiting endothelial-to-mesenchymal transition. Life Sci. 207:442–450. 29969608 10.1016/j.lfs.2018.06.033

[CIT0040] Sosulski ML, Gongora R, Feghali-Bostwick C, Lasky JA, Sanchez CG. 2017. Sirtuin 3 deregulation promotes pulmonary fibrosis. J Gerontol A Biol Sci Med Sci. 72(5):595–602. 27522058 10.1093/gerona/glw151 PMC5964739

[CIT0041] Srivastava SP, Li J, Kitada M, Fujita H, Yamada Y, Goodwin JE, Kanasaki K, Koya D. 2018. SIRT3 deficiency leads to induction of abnormal glycolysis in diabetic kidney with fibrosis. Cell Death Dis. 9(10):997. 30250024 10.1038/s41419-018-1057-0 PMC6155322

[CIT0042] Sun H, Che QM, Zhao X, Pu XP. 2010. Antifibrotic effects of chronic baicalein administration in a CCl_4_ liver fibrosis model in rats. Eur J Pharmacol. 631(1–3):53–60. 20079350 10.1016/j.ejphar.2010.01.002

[CIT0043] Sun X, Chen E, Dong R, Chen W, Hu Y. 2015. Nuclear factor (NF)-kappaB p65 regulates differentiation of human and mouse lung fibroblasts mediated by TGF-β. Life Sci. 122:8–14. 25498897 10.1016/j.lfs.2014.11.033

[CIT0044] Sun X, Cui X, Chen X, Jiang X. 2020. Baicalein alleviated TGF β1-induced type I collagen production in lung fibroblasts via downregulation of connective tissue growth factor. Biomed Pharmacother. 131:110744. 32932046 10.1016/j.biopha.2020.110744

[CIT0045] Sundaresan NR, Bindu S, Pillai VB, Samant S, Pan Y, Huang J-Y, Gupta M, Nagalingam RS, Wolfgeher D, Verdin E, et al. 2015. SIRT3 blocks aging-associated tissue fibrosis in mice by deacetylating and activating glycogen synthase kinase 3β. Mol Cell Biol. 36(5):678–692. 26667039 10.1128/MCB.00586-15 PMC4760222

[CIT0046] Takehara K, Koga Y, Hachisu Y, Utsugi M, Sawada Y, Saito Y, Yoshimi S, Yatomi M, Shin Y, Wakamatsu I, et al. 2022. Differential discontinuation profiles between pirfenidone and nintedanib in patients with idiopathic pulmonary fibrosis. Cells. 11(1):143. 35011705 10.3390/cells11010143 PMC8750555

[CIT0047] Tang LX, Wang B, Wu ZK. 2018. Aerobic exercise training alleviates renal injury by interfering with mitochondrial function in type-1 diabetic mice. Med Sci Monit. 24:9081–9089. 30551123 10.12659/MSM.912877 PMC6302662

[CIT0048] Teng C, Lin C, Huang F, Xing X, Chen S, Ye L, Azevedo HS, Xu C, Wu Z, Chen Z, et al. 2020. Intracellular codelivery of anti-inflammatory drug and anti-miR 155 to treat inflammatory disease. Acta Pharm Sin B. 10(8):1521–1533. 32963947 10.1016/j.apsb.2020.06.005 PMC7488359

[CIT0049] Tran BH, Yu Y, Chang L, Tan B, Jia W, Xiong Y, Dai T, Zhong R, Zhang W, Le VM, et al. 2019. A novel liposomal S-propargyl-cysteine: a sustained release of hydrogen sulfide reducing myocardial fibrosis via TGF-β1/Smad pathway. Int J Nanomedicine. 14:10061–10077. 31920303 10.2147/IJN.S216667 PMC6935304

[CIT0050] Tseliou E, Reich H, de Couto G, Terrovitis J, Sun B, Liu W, Marban E. 2014. Cardiospheres reverse adverse remodeling in chronic rat myocardial infarction: roles of soluble endoglin and Tgf-β signaling. Bas Res Cardiol. 109:443. 10.1007/s00395-014-0443-8 25245471

[CIT0051] Wang M, Dong Y, Wu J, Li H, Zhang Y, Fan S, Li D. 2020. Baicalein ameliorates ionizing radiation-induced injuries by rebalancing gut microbiota and inhibiting apoptosis. Life Sci. 261:118463. 32950576 10.1016/j.lfs.2020.118463

[CIT0052] Wang Q, Liu J, Hu Y, Pan T, Xu Y, Yu J, Xiong W, Zhou Q, Wang Y. 2021. Local administration of liposomal-based Srpx2 gene therapy reverses pulmonary fibrosis by blockading fibroblast-to-myofibroblast transition. Theranostics. 11(14):7110–7125. 34093874 10.7150/thno.61085 PMC8171094

[CIT0053] Wolters PJ, Collard HR, Jones KD. 2014. Pathogenesis of idiopathic pulmonary fibrosis. Annu Rev Pathol. 9:157–179. 24050627 10.1146/annurev-pathol-012513-104706 PMC4116429

[CIT0054] Wu W-H, Bonnet S, Shimauchi T, Toro V, Grobs Y, Romanet C, Bourgeois A, Vitry G, Omura J, Tremblay E, et al. 2022. Potential for inhibition of checkpoint kinases 1/2 in pulmonary fibrosis and secondary pulmonary hypertension. Thorax. 77(3):247–258. 34226205 10.1136/thoraxjnl-2021-217377

[CIT0055] Yang S, Wang H, Yang Y, Wang R, Wang Y, Wu C, Du G. 2019. Baicalein administered in the subacute phase ameliorates ischemia–reperfusion-induced brain injury by reducing neuroinflammation and neuronal damage. Biomed Pharmacother. 117:109102. 31228802 10.1016/j.biopha.2019.109102

[CIT0056] Yao Y, Chen R, Wang G, Zhang Y, Liu F. 2019. Exosomes derived from mesenchymal stem cells reverse EMT via TGF-β1/Smad pathway and promote repair of damaged endometrium. Stem Cell Res Ther. 10(1):225. 31358049 10.1186/s13287-019-1332-8 PMC6664513

[CIT0057] Zhang JX, Huang PJ, Wang DP, Yang WY, Lu J, Zhu Y, Meng XX, Wu X, Lin QH, Lv H, et al. 2021. m^6^A modification regulates lung fibroblast-to-myofibroblast transition through modulating KCNH6 mRNA translation. Mol Ther. 29(12):3436–3448. 34111558 10.1016/j.ymthe.2021.06.008 PMC8636177

