# Supplementary figures and images for: Baicalein attenuates bleomycin-induced lung fibroblast senescence and lung fibrosis through restoration of Sirt3 expression
Source: Pharm Biol. 2023 Feb 23;61(1):288–97. doi: 10.1080/13880209.2022.2160767 (PMC9970214; doi:10.1080/13880209.2022.2160767)

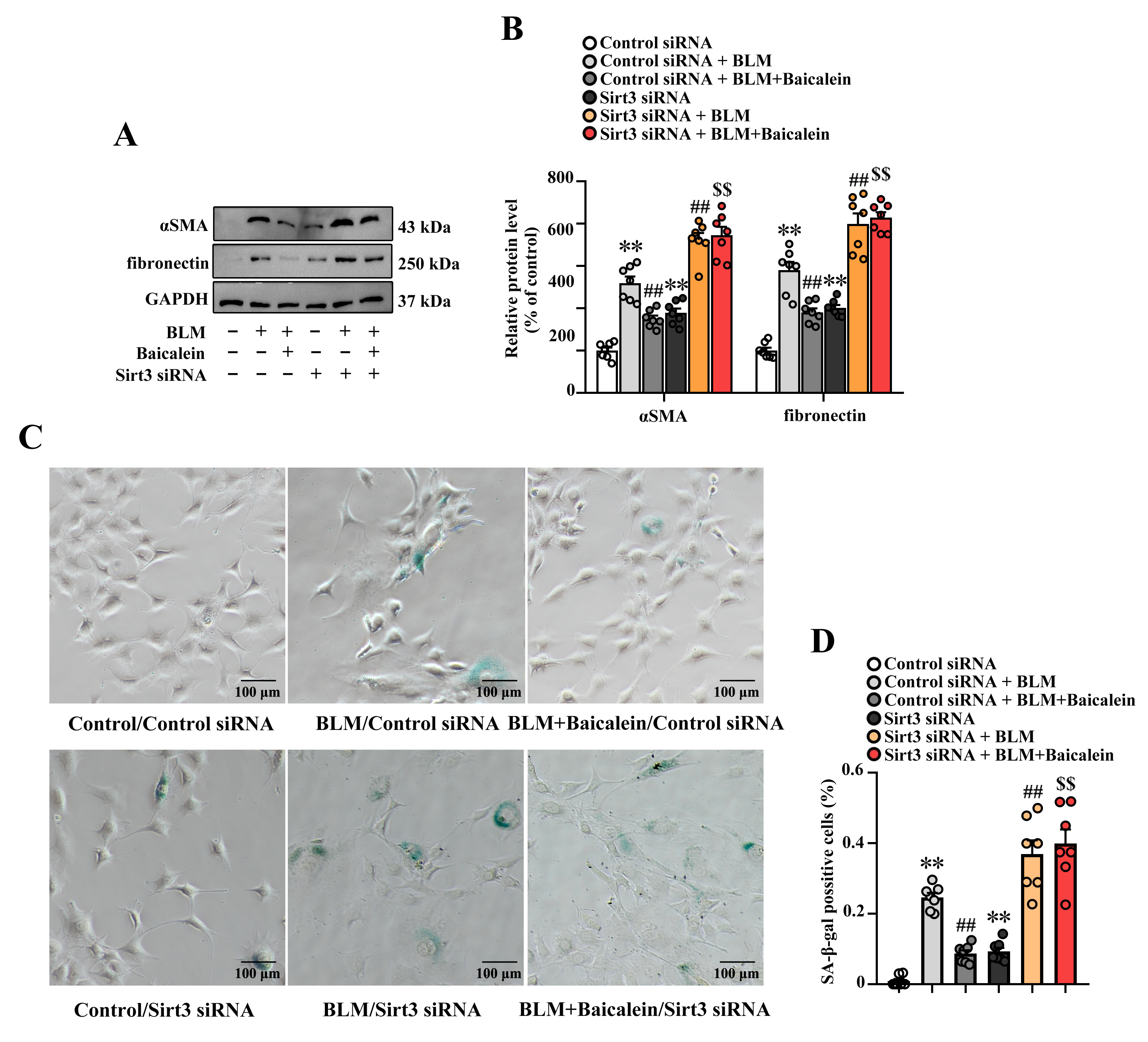

Supplement: Supplemental Material [file IPHB_A_2160767_SM9959.tif]

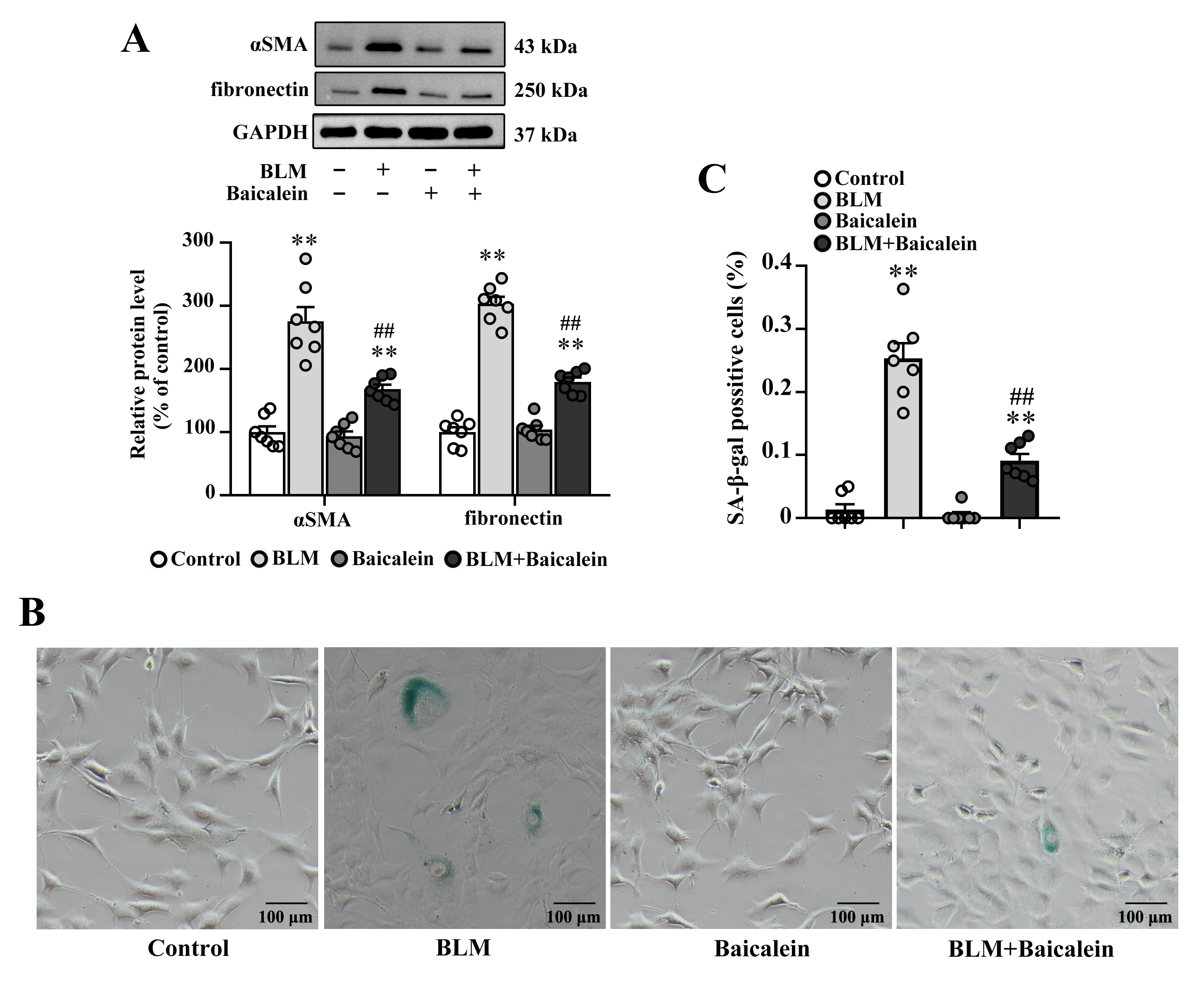

Supplement: Supplemental Material [file IPHB_A_2160767_SM9953.tif]
